# Monitoring electron energies during FLASH irradiations

**DOI:** 10.1088/1361-6560/abd672

**Published:** 2021-02-08

**Authors:** Alexander Berne, Kristoffer Petersson, Iain D C Tullis, Robert G Newman, Borivoj Vojnovic

**Affiliations:** 1 Medical Research Council Oxford Institute for Radiation Oncology, Department of Oncology, University of Oxford, Oxford OX3 7DQ, United Kingdom; 2 Radiation Physics, Department of Haematology, Oncology and Radiation Physics, Skåne University Hospital, Lund, Sweden

**Keywords:** FLASH, LINAC, radiobiology, electron beam energy, dosimetry, pre-clinical irradiation, pre-clinical radiotherapy

## Abstract

When relativistic electrons are used to irradiate tissues, such as during FLASH pre-clinical irradiations, the electron beam energy is one of the critical parameters that determine the dose distribution. Moreover, during such irradiations, linear accelerators (linacs) usually operate with significant beam loading, where a small change in the accelerator output current can lead to beam energy reduction. Optimisation of the tuning of the accelerator’s radio frequency system is often required. We describe here a robust, easy-to-use device for non-interceptive monitoring of potential variations in the electron beam energy during every linac macro-pulse of an irradiation run. Our approach monitors the accelerated electron fringe beam using two unbiased aluminium annular charge collection plates, positioned in the beam path and with apertures (5 cm in diameter) for the central beam. These plates are complemented by two thin annular screening plates to eliminate crosstalk and equalise the capacitances of the charge collection plates. The ratio of the charge picked up on the downstream collection plate to the sum of charges picked up on the both plates is sensitive to the beam energy and to changes in the energy spectrum shape. The energy sensitivity range is optimised to the investigated beam by the choice of thickness of the first plate. We present simulation and measurement data using electrons generated by a nominal 6 MeV energy linac as well as information on the design, the practical implementation and the use of this monitor.

## 1. Introduction

In recent years, the study of FLASH irradiation has attracted significant interest. A number of recent studies have demonstrated that irradiation at ultra-high dose rates (FLASH) diminishes the severity of toxicities in normal tissues compared to irradiation at the conventional dose rates (CONV) currently used in clinical practice (Favaudon *et al* 2014, Loo *et al* 2017, Montay-Gruel *et al* 2017, Bourhis *et al* 2019a, 2019b, Montay-Gruel *et al* 2019, Vozenin *et al* 2019, Wardman 2020). The mechanism responsible for reduced tissue toxicity following FLASH radiotherapy is yet to be elucidated and it is expected that both basic and pre-clinical work will continue with the aim of understanding the FLASH effect and applying the technique in clinical studies (Adrian *et al* 2020, Wilson *et al* 2020). Several publications have highlighted the difficulties associated with accurate dosimetry (Karsch *et al* 2012, Petersson *et al* 2017, Jorge *et al* 2019) when dose rates in the range of 30 Gy s^−1^—several MGy s^−1^, delivered in multiple or single pulses are used. Ion recombination effects in ionisation chambers generally preclude their use at these high dose rates (Boag 1950, Boag and Currant 1980, Gotz *et al* 2017). Hence, offline dosimeters like alanine and film are the preferred dosimeters (Fainstein *et al* 2000, Hayes *et al* 2000). To date, most preclinical work has been performed using electron beams generated by linear accelerators (linacs) of energies 4–20 MeV (Schüler *et al* 2017, Jaccard *et al* 2018, Lempart *et al* 2019). Due to limited penetration of such beams in tissues, beam energy is clearly one of the parameters of interest. The use of protons for FLASH irradiation (Patriarca *et al* 2018) has also been investigated and it may be possible to adapt the approach described here to such studies. A horizontal-firing linac, optimised for FLASH work is used in the work described here.

Maximising the output beam current is usually required for FLASH work, and the extent to which this can be achieved in a given linac design, assuming that beam current is available from the gun, is determined by the available radio frequency (RF) power and other accelerator design parameters, such as the shunt resistance and quality factor of the accelerating waveguide. The effects of beam loading on linacs (Burshtein and Voskresenskii 1963) are well-known; loading by the electron beam inevitably results in a drop of output energy and a change in the output spectral fluence. Two types of accelerating waveguide are commonly used, namely the standing wave (SW) and travelling wave (TW). In a SW linac, the RF power is fed to the accelerating waveguide using a power coupler which sets up standing electromagnetic fields in the accelerating waveguide and these fields are ultimately responsible for electron acceleration. In a TW linac, RF power is also fed to the accelerating waveguide using an input RF power coupler, which sets up a progressive electromagnetic wave in the accelerating waveguide to accelerate the electrons, and any power remaining at the end of the accelerating waveguide is dumped into a matched load, using an output RF power coupler. In a TW accelerator, as used in the setup described here, the RF power coupling into the accelerating waveguide is not affected by beam loading (Kulkarni *et al* 2016), unlike in the SW linac. Furthermore, in the TW type, the frequency of the RF drive can be varied according to the degree of beam loading (Arai *et al* 1984) to obtain the maximum energy gain and the minimum energy spread electron beam. In a heavily beam-loaded SW waveguide structure, the phase shift of accelerated electrons with respect to the accelerating field is significant, causing a drop in energy (Arai *et al* 1980). When the electron beam is bent through 90 or 270 degrees, as is commonly performed in medical linacs, usually with a quasi-achromatic magnetic deflector, some degree of energy selection is applied but often the output energy does indeed vary with beam loading. An on-line energy monitoring device is thus useful, particularly when the same accelerator is used for both high dose rate and low dose rate (CONV) irradiations. Moreover, it is useful to be able to return to particular previously used beam characteristics.

At the most basic level, beam energy can be determined using electron activation methods (Klevenhagen 1985). This exploits nuclear reactions that have threshold energies near to that of the energy of interest. This type of procedure is somewhat elaborate and therefore rarely used. Determining depth dose curves of electron beams obtained in water is the most commonly used method in the clinic (1984) to determine the energy-related parameters of an accelerator. This method uses the empirical relationships between the kinetic energy and range parameters of the penetration of the electrons in water.

Several methods have been employed in attempts to obtain details of electron spectra, which more directly reflect the distribution of incident electrons differential in energy, angle and position. These include electron scattering approaches (Blais and Padgorsak 1992) and spectral analysis approaches using magnetic deflection (Deasy *et al* 1994). Determinations from range measurements (Johnsen *et al* 1983) have also been used. One simple means of checking beam-energy by range measurement uses plastic scintillation fibres together with a copper wedge-shaped absorber; light emitted from the fibres is detected with photo-diodes (Aoyama *et al* 1995). Other ‘wedge’ methods exploit ionisation chambers for detection (Gao *et al* 2019). The Čerenkov method (Khupal 1973) has also been used for the measurement of the maximum incident electron energy only. Photon counting approaches (Blad *et al* 2002) have also been successfully implemented. However, all of these approaches intercept the beam and thus cannot be used during experiments or patient irradiations. While non-intercepting devices have been described in the literature, these require installation at the time of manufacture (Ruf *et al* 2014) or require fitting of additional cavities to the accelerating waveguide (Leggieri *et al* 2016).

Most of the above mentioned approaches and devices are relatively complex. A simpler approach that provides an indication of beam energy involves beam deposition on sequential aluminium plates, the thickness of which results in differential absorption of different energies in the plates (Fuochi *et al* 2003, 2005). This approach forms the basis for the energy monitor described here; however our device monitors the fringe beam and thus does not intercept the central portion of the beam, allowing it to be used continuously during irradiations. Each output macro-pulse during the irradiation can be monitored with our device. A device is described here that aims to monitor energy changes during an experiment, to return to particular previously used beam characteristics and not to obtain the energy spectrum per se. More complex versions of this approach using a series of ionisation chambers have been published (Geske 1990), and could be adapted for non-intercepting operation. However, these approaches not only require complex electronics but the use of ionisation chambers with high dose rate beams is not straightforward, as indicated earlier.

## 2. Experimental

### 2.1. Irradiation source and working distances

All experiments were carried out on our FLASH-optimised in-house developed 6 MeV nominal electron energy linac. This uses an Elekta SL75 TW waveguide, an S-band RF magnetron source (EEV-M5125 type, 2.89 GHz) and thyratron (CX1140)-based modulator, providing a repetitive beam of energetic electrons of ∼3.4 *μ*s pulse width, from a diode-type gun and original focusing magnets and supplies. All the accelerator control systems are bespoke, with pulse triggering performed by a phase-locked-loop with a 25 Hz reference (derived from 50 Hz ac mains) capable of providing pulse repetition rates in the range 25–300 Hz. All accelerator control and monitoring is provided through a PIC microcontroller via an optically isolated (duplex fibre) USB interface and bespoke software. The number of pulses being delivered in a session can be varied over a wide range and down to a single pulse, or the session can be allowed to run until a ‘stop button’ is pushed. The accelerator is arranged to fire horizontal beams through a thin output window (10 *μ*m thick beryllium-copper foil) into a temperature-controlled experimental area. The beam diameter at the window is usually ≈5 mm. Additional beam scattering is usually employed, provided by a titanium foil, 30 *μ*m thick, positioned 8.5 mm downstream from the window.

**Figure 1.**
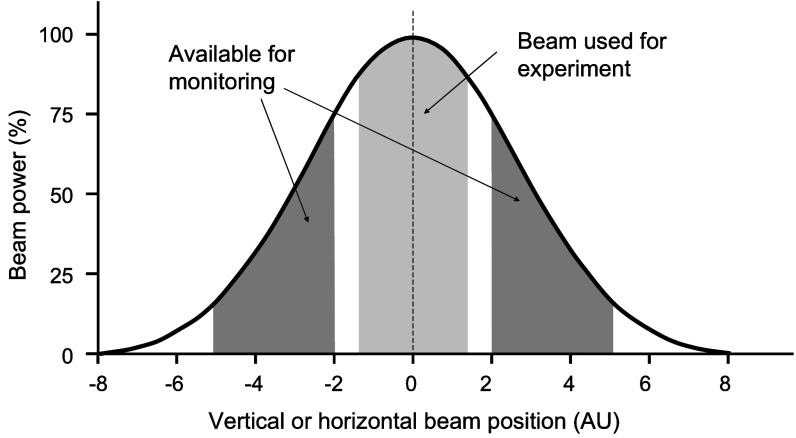
Application of non-intercepting energy monitor. Scattered electrons result in a Gaussian-like intensity distribution and only the central (flatter) portion is used for irradiating samples. The wings of the Gaussian are available for monitoring.

In the setup described here, the beam energy monitoring device is placed just in front of, and is mechanically connected to, a collimation arrangement that uses brass plates of 6 mm thickness, interchangeable so as to permit a range of output fields to be obtained. The irradiated sample is usually placed directly after this collimator. When using electron beams, dose at depth is significantly reduced when using beams of small cross sections, and the scattering properties of the window (along with any additional scattering foils placed in front of the window) are exploited to generate a typical beam profile such as that shown in figure 1. Usually relatively large beams (several centimetres in diameter) are generated in order to obtain a relatively flat profile in the central portions of the usable beam. The consequences of this are that a substantial portion of the fringe beam is available for monitoring, while the energy loss through scattering is acceptably low (Berger and Seltzer 1978, Patil *et al* 2011).

**Figure 2.**
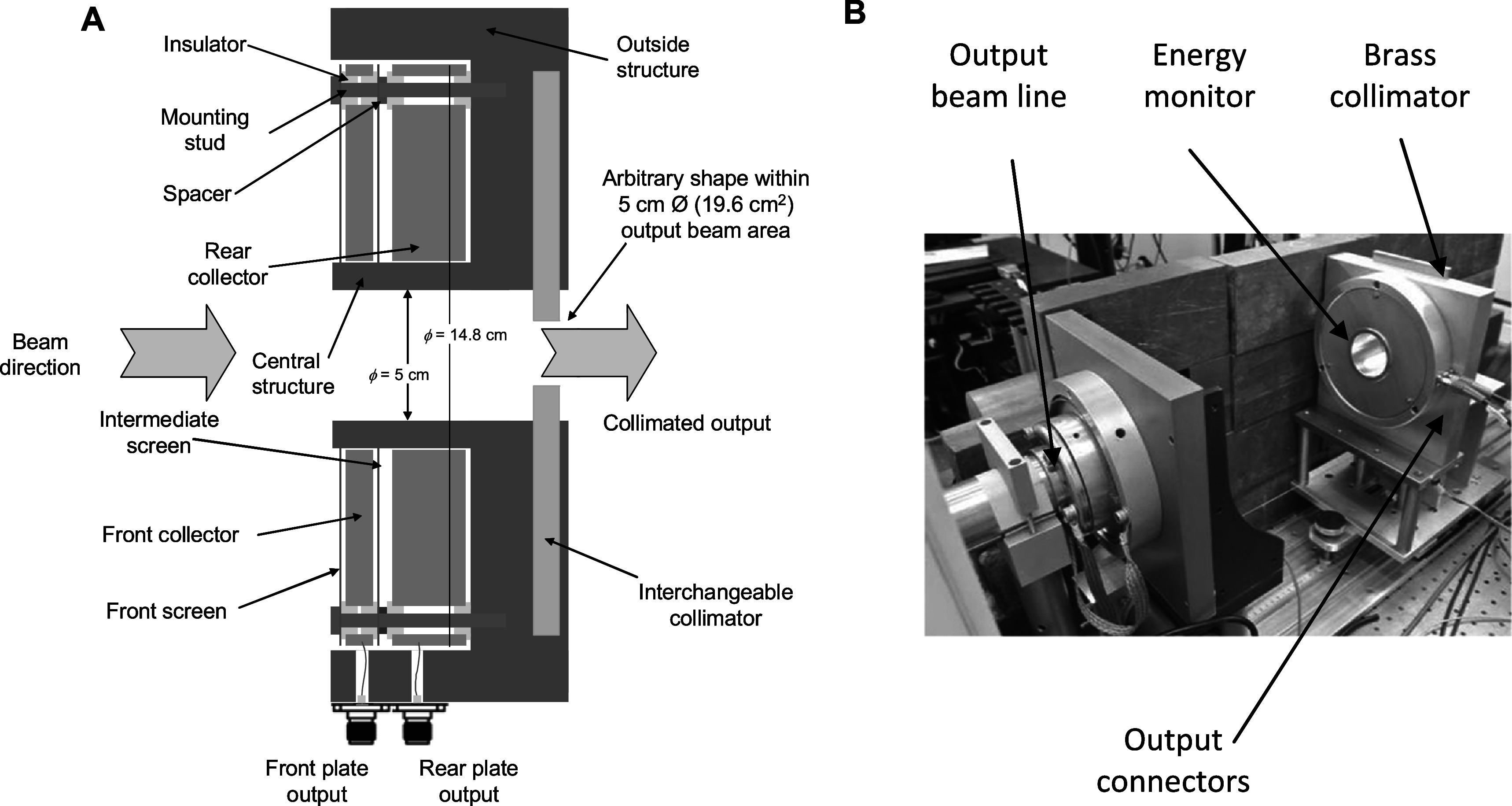
Panel A: Outline construction of the energy monitor and collimator assembly. Panel B: image of the completed energy monitor mounted on rails to allow variation of sample distance.

Our energy monitor and collimator arrangement is placed on rails allowing it to be moved away from the beam line window by up to ∼100 cm. The working distance (distance between the scatterer and the monitor) is measured with a string potentiometer type of sensor (Strainsense, Milton Keynes, UK, type CD80-2000-R01K-L15-L4). The construction and practical implementation of the energy monitoring device are shown in figure 2.

The energy monitoring device consists of two flat annular discs (collector plates) through which the non-intercepted central portion of the beam passes. These are surrounded by an earthed structure both outside and around the aperture, forming an imperfect Faraday cup type of arrangement placed coaxially with the beam path. The two collector plates are insulated from each other and from the Earthed structure. These two plates intercept the fringes of the beam.

The thickness of the front collector was selected according to the mean beam energy to be monitored, while the thickness of the rear collector plate is arranged to be thick enough to absorb all the fringe beam electrons, except at very large working distances where only part of the fringe beam is intercepted. The dimensions of all the components are shown in table 1. The separation between the screens and plates was 1 mm except between the second screen and the rear collector, where a separation of 3 mm was used. The separation between the rear collector plate and the outer structure was 1 mm.

**Table 1.** Dimensions of energy monitor.

Part	Outside diameter (mm)	Inside diameter (mm)	Thickness (mm)
Front screen	148.0	63.0	0.4
Front collector plate	148.0	63.0	6.0
Intermediate screen	148.0	63.0	0.4
Rear collector plate	148.0	63.0	16.0
Central structure	62.0	50.0	28.5
Outside structure	175.0	150.0	57.5

Since our linac delivers pulsed beams, collector–collector plate crosstalk and monitor output signal rise and fall times must be accounted for. As it is useful to monitor energy changes during the pulse(s), capacitive isolation between the plates should be ensured, or at least accounted for. Capacitive crosstalk between the intercepting plates has previously been investigated in a similar, albeit intercepting, device as the one described here (McChesney *et al* 2018). In our case, isolation is achieved by placing a thin (0.4 mm) screening plate between the collector plates, ensuring that any capacitive coupling between them is eliminated and allowing measurements to be made using different types of load on the collector plates.

Furthermore, the front collector plate presents a somewhat large area to any nearby interference sources. When performing measurements at very low dose rates, the beam charge picked up by the front plate can be as low as a few pico-coulombs and clearly potential interference could be problematic. A thin (0.4 mm) front screening plate is thus also fitted. The screening plate thicknesses are a compromise between mechanical rigidity and added beam scatter and attenuation.

It is not only useful to minimise collector plate capacitances to ground to ensure a fast response; it is useful to equalise the source capacitance from each collector plate to ground. This ensures that similar rise/fall times are obtained when the collector plates are loaded by similar resistances. This source capacitance similarity is ensured by increasing the gap between the second collector plate and the intermediate screening plate, achieved by placing a spacer on the mounting arrangement. All the charge collection plates are held by three M3 studs screwed into the Earthed body and the plates are insulated with ceramic washers. The front screening plate is held by the same studs and by outside M3 nuts. Finally, two SMA connectors are fitted at the side of the energy monitor and connected with short connections to the respective collector plates.

The calculated and measured capacitances were 285 ± 3 pF, limiting the minimum time constant to 14.3 ± 0.2 ns and the 10%–90% rise/fall times to 31.5 ± 0.5 ns into a 50 Ω load. Two lengths of 4.5 metre, 50 Ω terminated at a nearby recording instrument are used. These increase the measurement 10%–90% rise/fall times to 80.7 ± 1.4 ns, still acceptable for monitoring electron pulse widths of ∼3.4 *μ*s, particularly as the rise and fall times of the beam current, measured independently, are >150 ns. The plate and cable capacitances were determined by measuring the resonant frequency of a lightly loaded (5 MΩ) tuned circuit formed by a known-value inductor (203.5 *μ*H) and the capacitance of interest.

All components of the energy monitor components were constructed from aluminium, other than collimator (brass), mounting screws (steel) and insulators (ceramic). Aluminium was chosen because it is rugged, is readily machined, has a low backscattering coefficient (Ibbott 1985) as well as a high threshold energy for (*γ*, *n*) reactions (*E*_th_ = 13.1 MeV) (Varlamov *et al* 1967).

**Figure 3.**
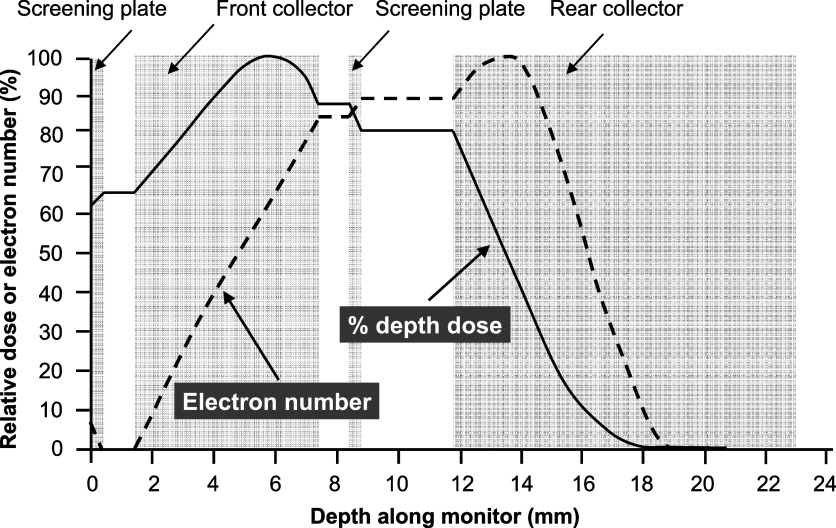
Representation of typical normalised percentage depth–dose curve distribution (solid line) and electron number distribution (dotted line) resulting from a 6 MeV mono-energetic electron beam interacting with the successive plates in the monitor, assuming negligible dose deposition and negligible electron loss in the air gaps between electrodes. The grey areas correspond to the collector plate positions. The electron beam enters from the left.

The thickness of the front collector ultimately determines the sensitivity of the device for different energies and was therefore chosen to match our accelerator’s beam energy. The aluminium thickness must be large enough such that a proportion of the incoming electrons is collected, as shown in figure 3. The practical electron beam range (*R*_*p*_) and the half-value depth (*R*_50_) in aluminium can be determined from such depth–dose distributions (ISO/ASTM 51649 2004). The percentage depth dose in aluminium is also presented in figure 3.

All plate charge measurements are of course imperfect as some charge is lost through secondary emissions, photon generation and other surface processes. Nevertheless, the charges collected on the front and the rear collector plates remain proportional to the beam current. All distances presented here are distances between the scatterer and the front of the first screen plate. These are referred to as ‘working distances’.

### 2.2. Energy determination and monitor positioning

Others have adopted the use of the term ‘energy ratio’ (Fuochi *et al* 2005) defined by:

\begin{eqnarray*}{{E}}_{{\mathrm{ratio}}}=\displaystyle \frac{{{Q}}_{{\mathrm{F}}}}{{{Q}}_{{\mathrm{F}}}+{{Q}}_{{\mathrm{R}}}},\end{eqnarray*}

where *Q*_F_ is the charge collected on front plate and *Q*_R_ is the charge collected on rear plate. The term *E*_read_ = *Q*_R_/(*Q*_F_ + *Q*_R_) has been adopted in this work since the value of *E*_read_ increases with energy and is thus a more intuitive indicator. In both cases, a beam pulse charge-independent (i.e. a ratiometric) measure of *E*_read_ is obtained.

**Figure 4.**
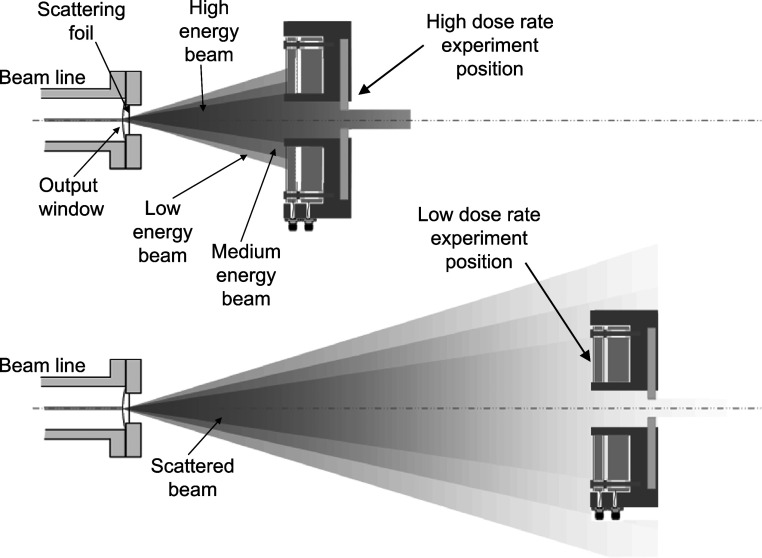
Diagrammatic representation of scattered beam output, of different experiment positions and portions of scattered beam available for experiment and for monitoring. A range of energies is produced by the scatterer (represented here as Low, Medium and High energy bands for simplicity), though in reality a continuous energy distribution is present. At the closest working distances the fringes of the higher energy components are not intercepted by the monitor.

**Figure 5.**
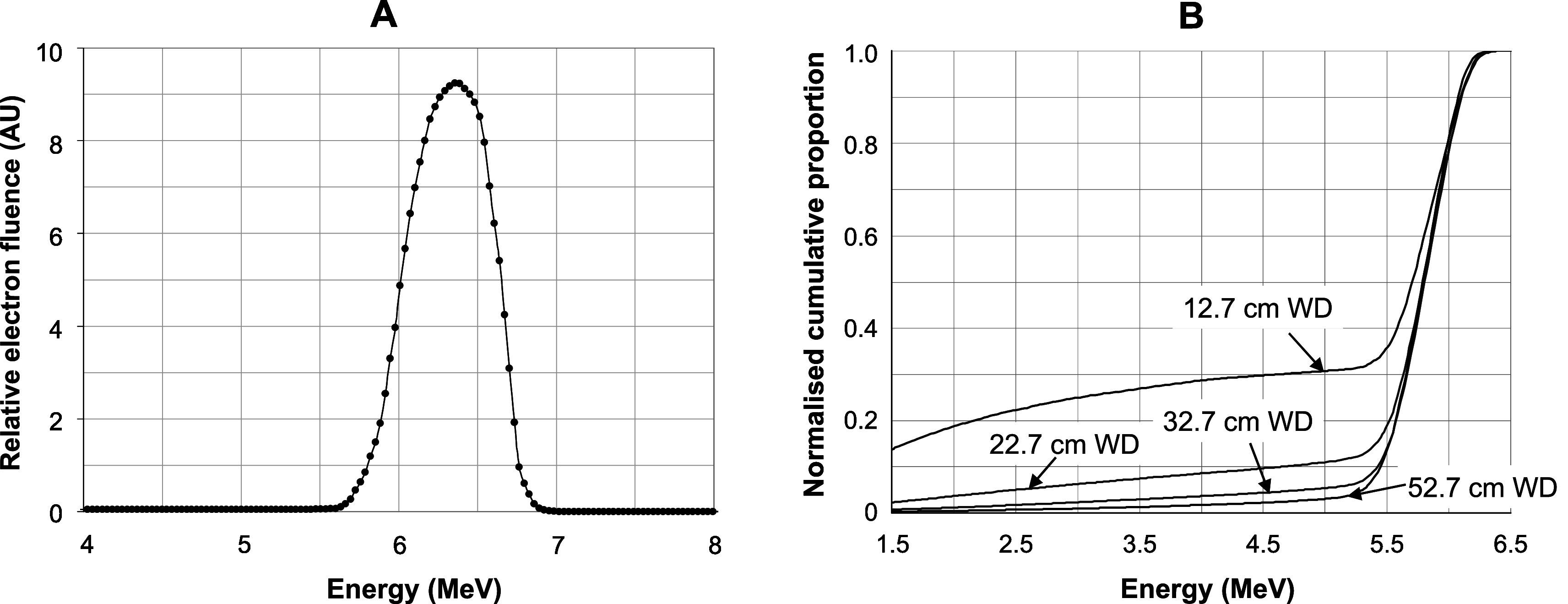
Left panel A: Simulated pre-window linac spectral fluence at near-maximum dose rate (FLASH dose rate) empirically determined through modelling of depth dose, lateral profiles and charge pick-up measurements over a range of working distances. Right panel B: Simulated energy degradation, due to the window and the scatterer, of the proportion of the beam intercepted by the energy monitor at different working distances. The plots show normalised cumulative spectra that indicate significant lower energy components are intercepted at close working distances. The cumulative spectra at longer working distances are similar to that at 52.7 cm working distance.

Beam energy variations result from variation of pulse width, electron gun emission and RF tuning. The *E*_read_ value also changes with variation of the distance between the scatterer and the experiment position. Changing the working distance inevitably causes different proportions of the lower energy scattered electrons to be picked up by the energy monitor, as shown in outline form in figure 4, while simulations are shown in figure 5 panel B. Both the beam line window and the scatterer will cause a shift of the energy fluence towards lower energies at larger angles. In addition, air between the scatterer and the energy monitor will further add to beam scatter and will broaden the spectral fluence. Air scatter is particularly significant at long working distances.

A range of calibration curves must therefore be determined at different working distances. Clearly, the device described here is a monitor, rather than an absolute measurement device. Its main purpose is to allow the linac operator to optimise beam parameters on-line and maintain a given (usually high) linac energy during the experiment at different working distances. These calibrations can be approximately derived through Monte Carlo particle simulations, where percentage depth–dose in phantoms, lateral profile and charge pickup measurements are used to derive the most likely input beam energy distributions.

### 2.3. Simulation

Monte Carlo simulation studies under idealised conditions have been performed in order to evaluate the performance of the energy monitor. All electron transport, dose, charge and energy simulations were performed with EGSnrc (nrc.canada.ca/en/research-development/products-services/software-applications/egsnrc-software-tool-model-radiation-transport and nrc-cnrc.github.io/EGSnrc/). The EGSnrc usercode ‘.cavity’ was used to simulate electron and photon transport. The minimum and maximum simulated energies for electrons (ECUT, EMAX) were set to 0.512 MeV and 50 MeV respectively. The minimum and maximum energies for photons (PCUT, PMAX) were set to 0.01 and 50 MeV. No variance reduction techniques were used for simulations. Other transport parameters were set to the default EGSnrc settings in the simulations.

Simulations were used to establish the expected spectral fluence of electrons impinging on the energy monitor. The output from any electron linac is far from being mono-energetic and this poses a fundamental problem when attempting to simulate the performance of the device. Simulations at different working distances have been performed, minimising differences between measurements and simulations of percentage depth dose, lateral profiles and charges collected at different working distances. We also simulated values of *E*_read_ (where *E*_read_ = *Q*_R_/(*Q*_F_ + *Q*_R_)) and of *Q*_T_ (where *Q*_T_ = *Q*_F_ + *Q*_R_) at different working distances. We further simulated beam charge collection in the central, non-intercepted region. The beam charge passing through the central hole was collected on a thick aluminium (50 mm) target.

We also simulated lateral profiles and depth dose measurements and compared these with measurements for a given spectral fluence over a range of beam divergences and found that these affected results when only unrealistic extreme divergence values were used (e.g. >±10°).

Air at standard conditions for temperature and pressure was assumed to be present around all components, except before the accelerator window, where vacuum was assumed. The uncertainty associated with the particle count was ≪1% in all simulations.

### 2.4. Charge readout

All electrical measured data were recorded as voltages on a Picoscope 4603 digitiser (Pico Technology, St Neots, Cambridgeshire, UK), converted to current by dividing by the digitiser’s 50 Ω input impedance and then converted to charge by integrating this current across the accelerator output pulse width. No significant DC offsets were observed that would interfere with such charge determinations. Charge integrations were performed using Matlab.

### 2.5. Beam measurements

Depth dose measurements were performed using solid water (15 × 15 cm^2^ rectangular slabs of RW3, PTW-Freiburg GmbH, Freiburg, Germany) and with radiochromic film (Gafchromic^TM^ EBT-XD, Ashland Inc., Covington, KY, USA) sandwiched between a series of 5 mm thick slabs. Lateral profile measurements were performed with radiochromic film positioned just in front of the energy monitor (in air). The films were read out with a film scanner (Epson Perfection v850 Pro, Seiko Epson Corporation, Nagano, Japan) and analysed with ImageJ (version 1.52a, Wayne Rasband, NIH, USA). The films were previously calibrated in a 6 MeV clinical electron beam from a Varian Truebeam (Varian Medical Systems Inc., Palo Alto, CA, USA) linac at the Churchill Hospital site in Oxford, UK.

## 3. Results

The simulated pre-window energy distribution derived as outlined in section 2.3 is shown in figure 5, panel A. Unfortunately, we did not have access to equipment to measure energy spectra but measurements of the beam lateral distributions and percentage depth dose at specific conditions allowed us to determine empirically the most likely spectral fluence. Numerous spectral fluence shapes were simulated until the differences between measured and simulated lateral profiles and percentage depth dose plots were minimised. A similar spectral fluence to the one derived here has been obtained by others (Johnsen *et al* 1983, McLaughlin *et al* 2018). The linac output window and the scatterer inevitably introduce lower energy components in the output spectrum, particularly at large scattering angles, as shown in figure 5 panel B. Increasing proportions of lower energy components are thus picked up by the monitor as the working distance is reduced.

**Figure 6.**
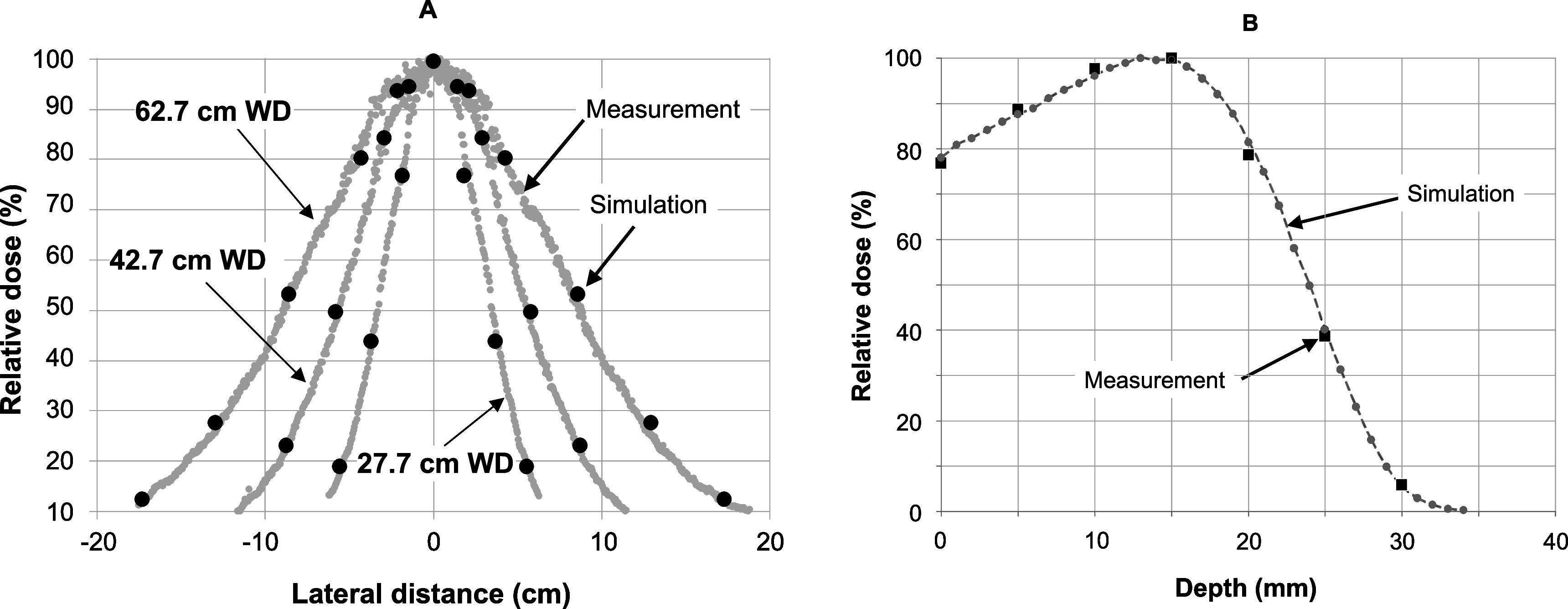
Left: Comparison of measured (solid grey lines) and simulated (round black points) lateral profiles at 27.7, 42.7 and 62.7 cm working distances, showing acceptable agreement between acquired and simulated data sets. Right: comparison of measured (solid squares) and simulated (dotted lines and round points) percentage depth dose data in a solid water phantom at a working distance of 62.7 cm.

**Figure 7.**
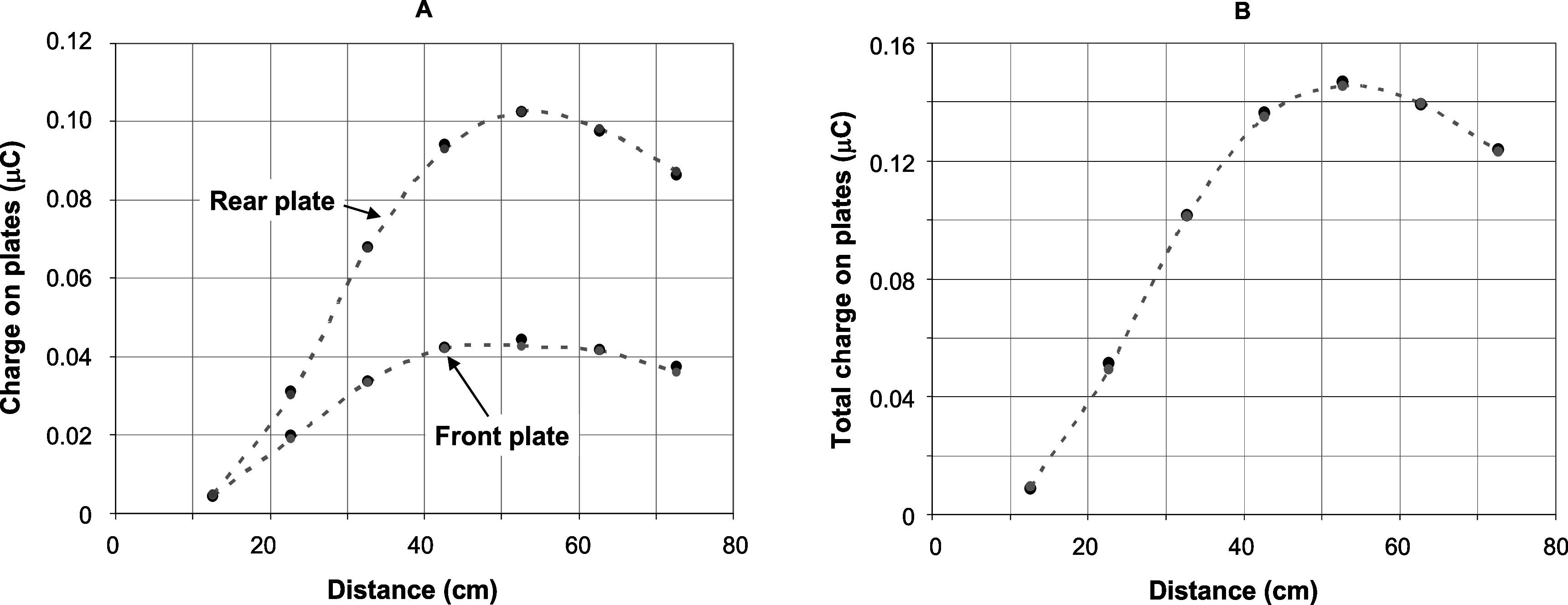
Panel A: Comparison of measured charges on the front and rear plates (solid round black points) and simulated charge pickup on the plates (small grey points and dashed lines). Panel B: Comparison of sum of measured charges on both plates, *Q*_T_, (solid round black points) and sum of simulated charges (small grey points and dashed lines). A reasonable agreement (<±3%) is obtained between measurement and simulation.

**Figure 8.**
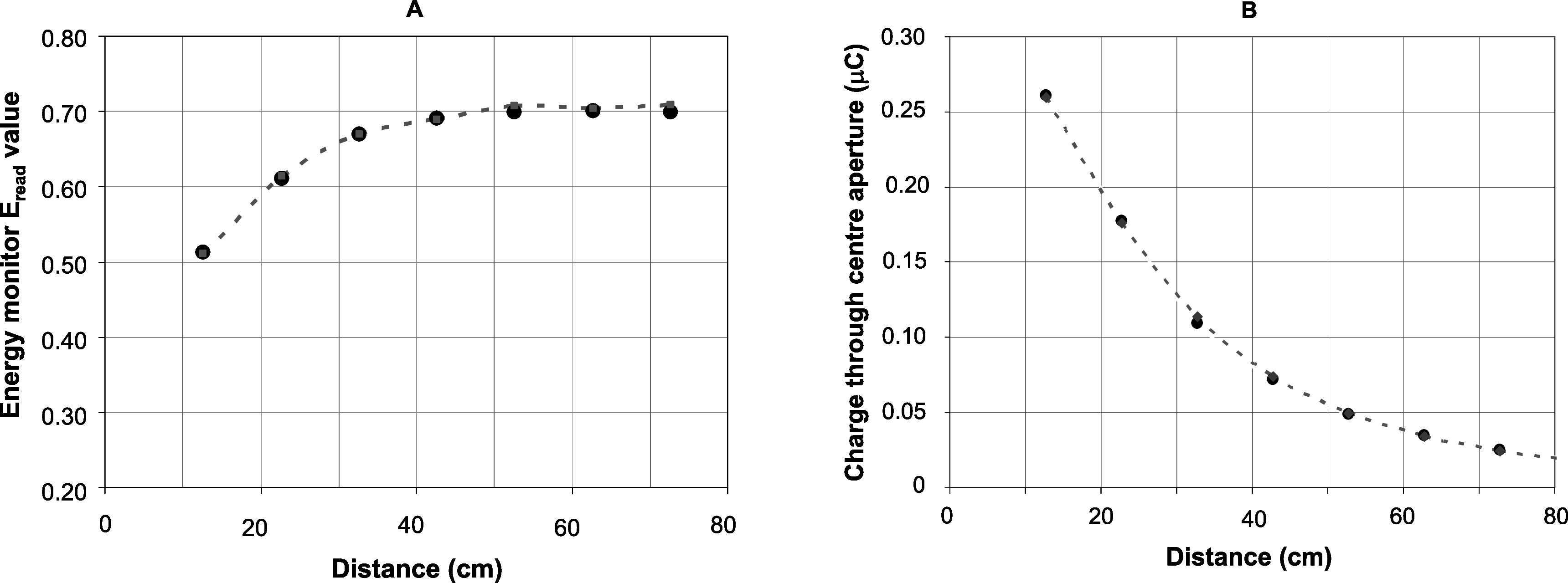
Panel A: Comparison between measured (solid round black points) and simulated (small grey points and dashed line) ratio of rear plate charge to the sum of charges on both plates as a function of distance from scatterer. This charge ratio is the readout of the energy monitor. Panel B: comparison of measured (solid round black points) and simulated (small grey points and dashed line) charge passing through the central aperture as a function of working distance.

Simulated lateral dose profiles (in air) at different working distances were compared with measured lateral dose profiles. Furthermore, a percentage depth dose measurement at a working distance of 62.7 cm was determined and compared with simulations. These data are presented in figure 6, and show good agreement between measurements and simulations using the spectrum presented in figure 5, panel A. Comparisons between acquired and simulated charge measurements, as shown in figures 7 and 8 also show good agreement. However, all the data presented in figures 5–8 do show some slight differences, well within ±3%, between measured and simulated data. This can be due to several factors: (1) the pre-window electron beam is assumed to be collimated, when in reality some slight convergence of the beam is likely to exist due to the use of an upstream focusing magnet, some 1.6 m away from the output window; (2) the beam may not be truly coaxial with the energy monitor, though every effort has been made to make it so; (3) the beam cross-section may not be perfectly symmetrical; (4) the charge collectors act as imperfect collectors without the usual ‘deep’ cup hole used in Faraday cups (5) at longer working distances, backscatter from additional structures that are not readily simulated will influence the charge collection by the monitor. These structures include the monitor base plate and rails, an optical table onto which the rails are mounted and a lead wall on one side of the monitor rails, ∼25 cm away from the beam axis. Although 3D models of these structures were available, transfer of these data into EGSnrc was not possible. Since these structures were located relatively far from the beam axis we only simulated the performance of the monitor in its housing. The simulation results were linearly scaled, with no offset, and overlaid onto measured data.

**Figure 9.**
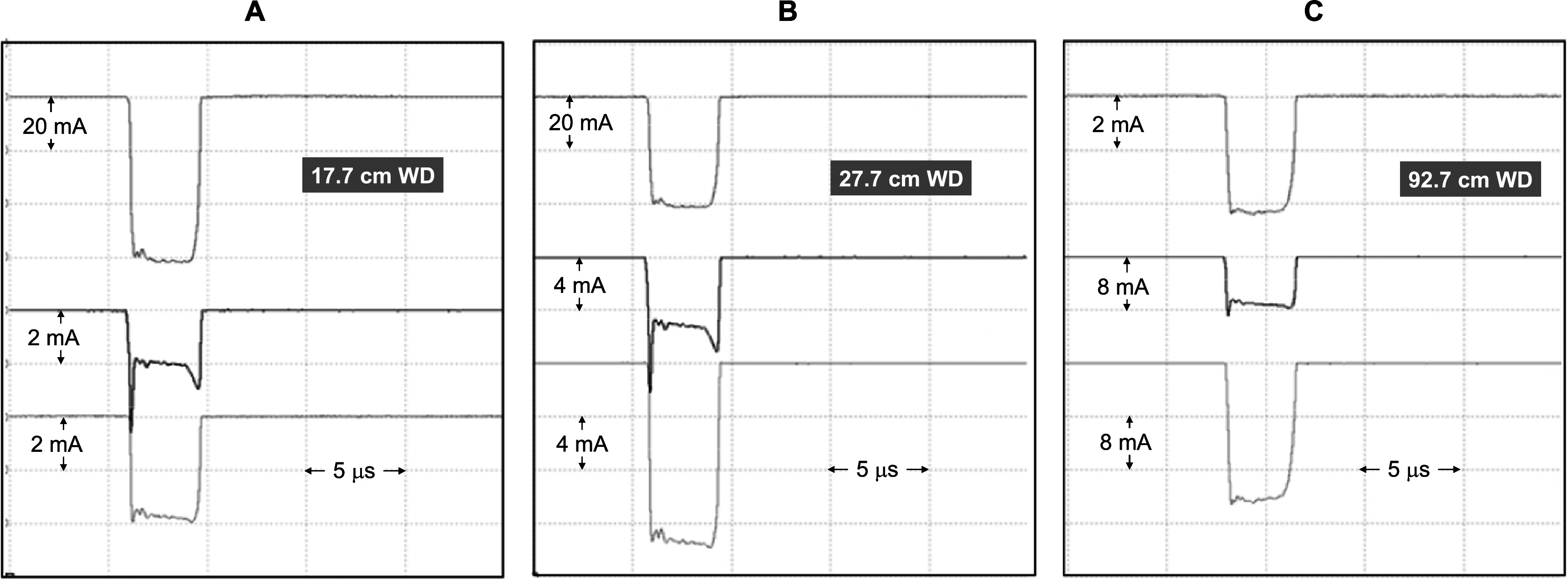
Representative charge pulses collected through the central aperture (upper traces) the front plate (middle traces) and the rear plate (lower traces) at working distances of 17.7 cm (panel A) 27.7 cm (panel B) and 92.7 cm (panel C). Measurement performed with 50 Ω loads over a bandwidth of DC − 5 MHz.

At a long working distance, most of the low energy components from the linac output have been scattered out of the beam capture range of the finite diameter collection plates of the energy monitor. This is clearly shown in the oscillograms in figure 9: an increase in current collected by the front plate represents an increase of the low energy components collected. These are present at the shorter working distances at the start and the end of the pulse but are not prominent at long working distances. At the start of the pulse not all the electrons have been correctly bunched in the linac while at longer times, the linac modulator’s pulse forming network output voltage starts to decay.

**Figure 10.**
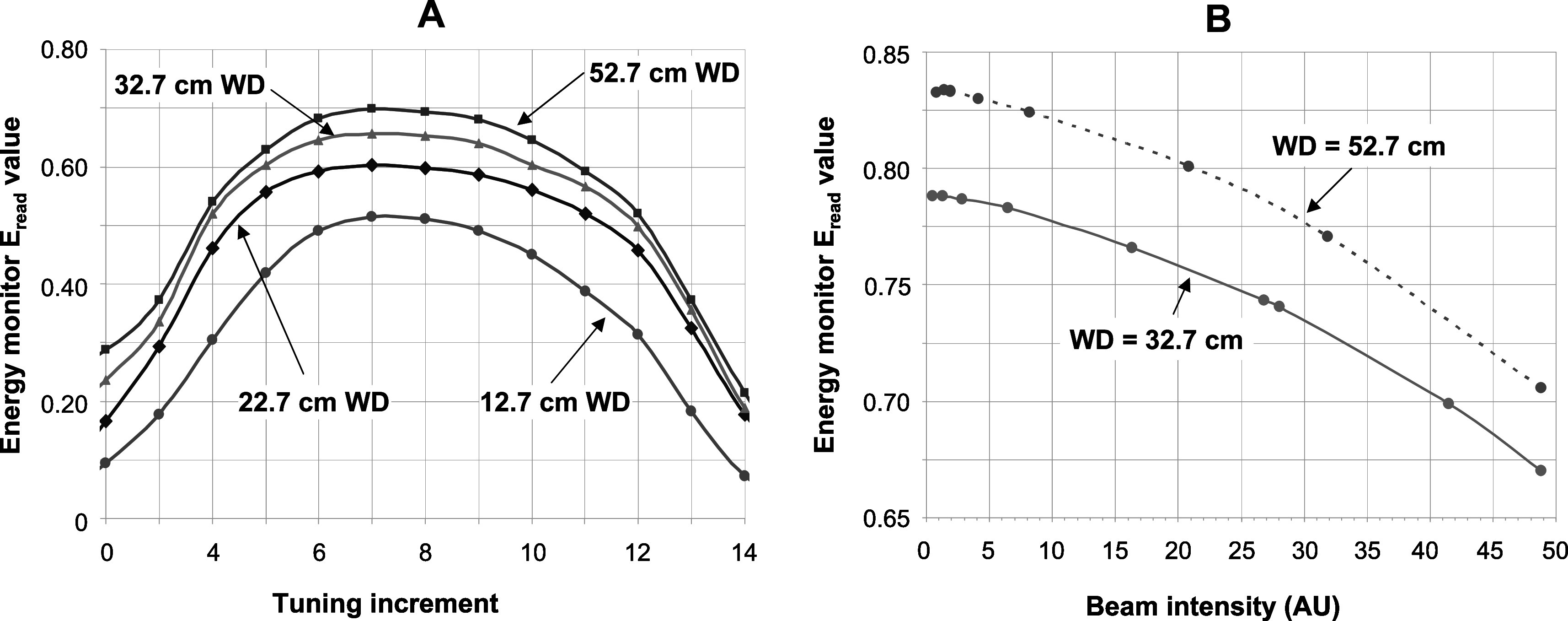
Panel A: Energy monitor *E*_read_ plotted as a function of ∼40 kHz magnetron tuning increments for several scatterer-monitor distances. Tuning responses at longer distances are similar to that acquired at 52.7 cm. Panel B: Plot of *E*_read_ as a function of output beam intensity, modified by changing injection currents, and demonstrating the consequences of beam loading in the accelerator.

Nevertheless, the plot in figure 8, panel A is considered to be an appropriate output measure of the overall beam energy distribution. It is this measure that is maximised when the accelerator’s RF frequency is adjusted in order to maximise the beam energy. The variation in this measure with RF tuning as a function of distance is shown in figure 10 panel A. Here, the linac magnetron is tuned in the smallest frequency steps available to us (each step corresponding to ∼40 kHz change in the 2.998 GHz magnetron frequency). This plot shows that the optimum tuning point to provide the most energetic electron output can be easily determined. It also shows that accelerator tuning is best performed at distances at which the collection plates are able to sense the largest proportion of the scattered beam (WD ≈ 50 cm). At very long distances, the measured *E*_read_ output drops slightly because of air scatter and due backscatter from other structures on either side of the monitor. These structures were not part of the simulations performed. It is noted that when the magnetron frequency was significantly detuned, well past the extremes of the tuning curve shown in figure 10(A), unstable machine operation is inevitable and must be avoided.

Furthermore, as the beam current is reduced, the average linac energy will inevitably increase. Variations of *E*_read_ at different working distances and beam currents are shown in figure 10 panel B. Output beam current reduction was achieved by lowering the gun filament temperature by reducing current flowing through the gun filament. Approximate conversion of *E*_read_ values to beam energy can be deduced from data presented in supplementary figure 1 (available online at stacks.iop.org/PMB/66/045015/mmedia) where the monitor’s response to mono-energetic beams is presented.

It is noted that when the accelerator’s RF system is optimally tuned for FLASH irradiation, there is negligible variation of monitored energy with time or with number of pulses delivered, and the output energy is centred at 6.35 MeV. We did not measure the energy drop as a function of peak pulse current, but as noted in figure 10(B), the energy can increase significantly during operation at conventional dose rates, up to ∼7.5 MeV or more.

## 4. Discussion

A simple and effective monitoring device that can provide on-line monitoring of electron beam energies delivered by a 6 MeV nominal energy linac has been described. The details of the energy monitor construction are provided, and typical data that can be acquired with it are presented. The device can provide: (1) an indication of the most likely electron pulse energy in near real-time; (2) indications of any variations in that energy during potentially unstable accelerator operation; (3) operation over a wide range of pulse charges, corresponding to those required for FLASH irradiations as well as conventional, low dose rate, irradiations and (4) a straightforward means of tuning the accelerator to provide specific energies. Simulations have been performed to validate the performance of the energy monitor and although these only fit measured data to within <±3%, it is unlikely that this can be significantly improved upon using an empirically determined linac output spectrum. Means to determine the electron spectral fluence independently were not available; instead it was postulated that our linac is likely to deliver a spectrum whose FWHM would be similar to output spectra of similar machines. In our simulations, the mean energy was adjusted and slight changes to the spectrum shape were made in order to minimise differences between measured and simulated data representing lateral profiles, percentage depth–dose data (figure 6), charges collected by the monitor plates and to charges passing through the central aperture (figures 7 and 8). The device sensitivity is further exemplified in figure 10 which confirms that the linac can be readily tuned to provide the maximum energy used during FLASH irradiations. Alternatively, when performing CONV dose rate irradiations, the machine can be detuned to provide energies comparable to those used during FLASH irradiations, by using low output current intensities that would otherwise result in higher energies due to reduced beam loading. In practice the energy monitor described here is most useful when irradiating samples over a large dose rate range, spanning FLASH dose rates (ranging from a few MGy s^−1^ down to some tens of Gy s^−1^) and all the way down to CONV dose rates of a few Gy min^−1^. The energy monitor can be used to modify the accelerator tuning in a repeatable fashion to previously used beam energies. Specific energies cannot be selected on our accelerator and it was thus not straightforward to determine the sensitivity of the monitor to typical beams over a range of output energies. However, we have simulated the monitor’s response to mono energetic simulated beams, as shown in the supplementary material in figure S1. The sensitivity to energy is nonlinear, as was expected. The *E*_read_ values (at a working distance of 52.7 cm) varies from 0.17 to 0.49 units between 4 and 5 MeV, from 0.49 to 0.7 units between 5 and 6 MeV, from 0.7 to 0.83 units between 6 and 7 MeV, from 0.83 to 0.89 units between 7 and 8 MeV and from 0.89 to 0.93 units between 8 and 9 MeV; this nonlinear response clearly shows enhanced sensitivity for 6 MeV and lower energies, as is appropriate for our accelerator.

The same principle described here can be exploited for monitoring energy changes in more energetic linacs or of course less energetic ones. The front plate thickness must be increased in the former case and decreased in the latter case. Although the design can be optimised, when electron energies significantly above 20 MeV are used, or large field irradiations are performed, the weight of the plates may limit its usefulness and appropriate means of positioning will be required. Furthermore the device central aperture diameter and outside plate diameter should be modified in order to suit the collimation used. While we do not claim that the device described here can be systematically used in all FLASH set-ups, we trust that it can be suitably optimised as required.

Simulation of the output from inherently mono-energetic machines such as electron Van de Graaffs or Rhodotrons is simpler as significantly lower variations in *E*_read_ with working distance will be obtained. Simulations shown in supplementary figure 1 have also been performed to establish the sensitivity of *E*_read_ to beam energy. These show that that the *E*_read_ ratio does provide a high sensitivity to mono-energetic beam energy changes. It is however noted that the *E*_read_ ratio does not reflect the beam energy that impinges on the sample, which is usually irradiated with a slightly higher energy. The *E*_read_ ratio only indicates the mix of energies from the scattered primary beam that is sampled by the monitor plates. It is assumed that the linac spectral fluence, when the linac is operating at a given pulse current, does not change. Irradiations performed at lower peak currents and at longer distances are always backed up by percentage depth dose measurements at these different conditions. This is why this device is an energy monitor rather than an energy meter. An intercepting monitor, constructed in a similar manner, would indeed be able to ‘measure’ the beam energy.

The charges collected on the monitor output plates can be measured using any established charge measurement approach. Additional details of signal acquisition and signal processing are presented in the supplementary data. When using an oscilloscope input and a 50 Ω-terminated interconnecting cable, and a single macro-pulse widths of ∼3.4 *μ*s, charges of the range of 100 pC (corresponding to ∼−30 *μ*A peak current) to 150 nC (corresponding to ∼−45 mA peak current) can be readily acquired. Indication of temporal changes in energy can be acquired over this range.

Should a lower pulse width be used, lower charges/pulse can be monitored but the plate and cable capacitance must be evaluated and reduced as necessary. Of course, if the oscilloscope input impedance is increased to 1 MΩ then a leaky integrator is formed and much more sensitive measurements can be performed. This approach can be used to monitor significantly lower total charges/pulse (down to a few pico-coulombs) but the ability to view intra-pulse changes is then lost. An average energy reading (*E*_read_) during a multi-pulse irradiation sequence can also be monitored if required; however, use of very low leakage integrators then becomes essential.

Various approaches for signal acquisition, including the use of a bespoke integrator, coupled to a readout system are described in the supplementary material. We prefer to monitor the energy pulse-by-pulse; this provides us with statistical measures of potential beam energy variations.

## 5. Conclusion

A simple, dose-rate independent device for monitoring energies of electron beams used for FLASH irradiation has been presented here. The device response has been analysed using Monte-Carlo approaches and has been validated with experimental data acquired with a pulsed electron beam delivered from a linear accelerator. The energy monitor has been found to be particularly useful during accelerator tuning procedures. Since it monitors the fringes of the beam used during irradiations of pre-clinical samples, it can be used during FLASH and CONV dose rate irradiations. It can provide a real-time readout of beam energy variations, should they occur due to excessive beam loading as can all too easily occur at very high FLASH dose rates. Furthermore, the device described can confirm, in real time, the energy associated with a given irradiation, whether this is a FLASH or CONV irradiation. This permits biological straightforward comparisons to be performed between FLASH and CONV, ensuring that comparable energy deposition at depth is obtained in the two cases. Although the FLASH effect is not dependent on energy, it is dependent on dose and the dose to deeper tissues is significantly reduced during low energy irradiations. This is an inherent limitation of using low MeV electron beams for FLASH work. Our monitor therefore plays a useful role in quality assurance both prior to and during pre-clinical irradiations.
